# Community violence exposure and mental health in urban Mexico: The moderating role of school belonging in early adolescence and young adulthood

**DOI:** 10.1111/jora.70235

**Published:** 2026-07-26

**Authors:** Andrea S. Medrano, Patrick T. Quintero, Erika Álvarez Álvarez, Francheska Alers‐Rojas

**Affiliations:** ^1^ Department of Psychology University of Pittsburgh Pittsburgh Pennsylvania USA; ^2^ Department of Psychology Universidad Michoacána de San Nicolás de Hidalgo Uruapan Mexico; ^3^ Department of Human Development & Family Sciences University of Texas at Austin Austin Texas USA

**Keywords:** community violence exposure, mental health, Mexico, school belonging

## Abstract

Community violence exposure (CVE) is a pervasive risk to youth mental health, yet developmental research examining protective factors in Latin America remains scarce. This cross‐sectional investigation examined CVE and psychological symptoms among early adolescents (*N* = 346) and young adults (*N* = 499) living in a chronically violent region of Michoacán, Mexico. Participants completed validated measures of witnessing violence, personal victimization, school belonging, depressive symptoms, and posttraumatic stress symptoms (PTSS). Results indicated that CVE was widespread in both age groups and consistently associated with elevated depressive symptoms and PTSS. Notably, developmentally distinct moderation patterns emerged. Among early adolescents, higher school belonging intensified the associations between CVE and depressive symptoms, consistent with a vulnerable‐reactive pattern. In contrast, school belonging buffered the association between CVE and PTSS among young adults. These findings suggest that the function of school belonging may differ by developmental stage, operating as a source of potential ecological sensitivity in early adolescence and as a resilience resource in young adulthood. Results underscore the importance of developmentally tailored supports for youth navigating chronic violence in the Global South.

## INTRODUCTION

Community violence exposure (CVE) is a pervasive global public health concern, affecting youth across diverse national contexts and carrying well‐documented consequences for mental health, particularly depressive symptoms and posttraumatic stress symptoms (PTSS; Fowler et al., 2009; World Health Organization, 2020). Despite decades of research documenting these risks and more recent literature focusing on potential protective factors (Miliauskas et al., 2022; Mora et al., 2024; Dill & Ozer, 2016), much of the literature has centered on communities in the Global North. In contrast, Latin America and the Caribbean represent one of the most violence‐affected regions globally, with youth bearing a disproportionate burden of exposure (UNICEF, 2022). Within this broader context, several cities in Michoacán, Mexico have consistently ranked among the most violent cities worldwide due to organized crime and armed conflict (Cure Violence Global, 2023). These conditions provide a critical context for examining how CVE operates across development. The current study therefore investigates whether school belonging functions as a protective factor in the link between community violence and mental health, as assessed by depressive symptoms and PTSS. We further examine whether these links function differentially for early adolescents and young adults in the context of one high‐violence city in Michoacán. In doing so, the present study seeks to understand age‐related differences to inform developmentally grounded strategies that promote psychological well‐being among Mexican youth navigating chronic exposure to community violence.

### Community Violence Exposure (CVE)

Community violence exposure (CVE) refers to indirect (witnessing violence) or direct (personal victimization) experiences of intentional acts of violence occurring outside the home (Ceballo et al., 2022). In Mexico, such exposure is widespread. National data indicate that over 60% of Mexican youth aged 12–29 report witnessing community violence and nearly 30% reported being personally victimized (Pérez‐Sastré et al., 2024b), with even higher rates documented in high‐risk regions such as Michoacán. For instance, Méndez‐López and Pereda (2019) found that 61% of adolescents (*N* = 1068) had witnessed or been personally victimized by violence in their lifetime, with 45% reporting such exposure in the past year. Together, these findings underscore both the regional salience of violence in Michoacán and the chronic, widespread nature of violence exposure among Mexican youth.

Despite the prevalence of CVE, less is known about how these exposures vary across developmental stages. Regional data show that violence peaks during adolescence in Latin America and the Caribbean (United Nations International Children's Emergency Fund [UNICEF], 2022). These data reveal that the child and adolescent homicide rate in this region is four times higher than the global average, with homicide being the leading cause of death among youth ages 10–19 (UNICEF, 2022). National homicide trends in Mexico indicate that adolescents become increasingly vulnerable to community violence in mid‐ adolescence (López‐Ortiz et al., 2024). Research also finds that youth between ages 15 and 19 experience some of the highest rates of violence exposure, reflecting structural and social conditions that place adolescents at heightened risk (UNICEF, 2022). Indeed, López‐Ortiz et al. (2024) found that adolescents aged 15–19 experience sharply elevated homicide rates in Mexico, with 32.45 per 100,000 among boys and 5.65 per 100,000 among girls.

More specifically, adolescent boys and young men bear the greatest burden of lethal community violence overall, representing 88.96 percent of all homicide deaths, with over 60 percent killed by firearms in public spaces such as streets and highways. López‐Ortiz et al. (2024) further found that the odds of death in adolescence were nearly 19 times higher for boys and 7 times higher for girls, respectively, compared with early childhood. Taken together, these patterns suggest that both the frequency and severity of community violence escalate from early adolescence into early adulthood, underscoring the need to examine CVE as a dynamic developmental process rather than a uniform experience. Yet, most existing research focuses on school‐aged adolescents, leaving young adulthood comparatively understudied and limiting opportunities to identify how risk may intensify or manifest differently across developmental periods (Méndez‐López & Pereda, 2019; Santacrose et al., 2021). To address this gap, the present study examines CVE patterns in early adolescence and young adulthood to illuminate these developmental differences.

### Community Violence Exposure (CVE) and Psychological Outcomes

A substantial body of research has documented the links between CVE and worse mental health outcomes (Benjet et al., 2009; Fowler et al., 2009; McDonald & Richmond, 2008; Miliauskas et al., 2022; Mrug & Windle, 2010; Santacrose et al., 2021). Although personal victimization is often speculated to confer the most risk for developing mental health disorders, witnessing violence or living in neighborhoods marked by persistent violence can, in some cases, predict even more severe mental health symptoms, including PTSS and, to a lesser extent, internalizing symptoms such as depression (Fowler et al., 2009). Notably, meta‐analytic evidence suggests that Latino youth may be particularly vulnerable to PTSS following CVE exposure, showing stronger associations between violence exposure and posttraumatic stress than other groups (Fowler et al., 2009). Indeed, findings from the World Mental Health Surveys in five Latin American cities (*N* = 7,251) show that adults living in neighborhoods with higher violence had greater odds of past‐year mood and anxiety disorders (Benjet et al., 2019). Among Mexican adolescents specifically, personal victimization has been associated with significantly elevated depressive symptoms (Balmori‐de‐la‐Miyar et al., 2025). Yet, the psychological burden of community violence is not limited to those who are directly targeted—witnessing violence can also create persistent uncertainty and anticipatory threats to youth's sense of safety (McDonald & Richmond, 2008). Consistent with a poly‐victimization framework, CVE often reflects not a single isolated event but co‐occurring experiences across multiple forms of violence (Finkelhor et al., 2007). In the current study, we capture this breadth by examining both specific forms of exposure and broader domains of witnessing violence and personal victimization, allowing us to characterize the range and pervasiveness of exposure across developmental stages.

While prior research has often examined mental health outcomes collectively under an internalizing umbrella (Benjet et al., 2019), the present study treats depressive symptoms and PTSS as conceptually distinct outcomes. Depressive symptoms reflect broader disruptions to mood, motivation, and social functioning that accumulate through chronic stress, erosion of perceived control, and loss of trust in one's social environment (Hammen, 2005). PTSS, by contrast, reflect threat‐based responses including hypervigilance, avoidance, and intrusive re‐experiencing that are closely tied to perceived danger and unpredictable exposure to violence (Ehlers & Clark, 2000). This distinction is important because the processes linking CVE to depressive symptoms may differ from those underlying PTSS, suggesting that protective factors such as school belonging may not operate uniformly across outcomes.

Exposure to community violence at distinct developmental stages may affect psychological health differentially. Adolescence represents a particularly sensitive period of development because youth are undergoing major biological, cognitive, and socioemotional transitions (Steinberg, 2014). These transitions involve consolidating identity, strengthening coping strategies, and adapting to increasing exposure to stress and environmental demands, which heightens vulnerability to adverse contexts such as community violence (Cicchetti & Toth, 2009; Steinberg, 2005). Given that nearly half of lifetime mental health disorders have their onset in early adolescence (Solmi et al., 2022), exposure to risks such as community violence during this time can further heighten vulnerability to developing mental health problems (e.g., anxiety & depression). By contrast, some youth ages 19–29 exhibit fewer distress symptoms when living in high‐criminality areas (Pérez‐Sastré et al., 2024a). This unexpected inverse connection may reflect adaptation, normalization, or greater coping strategies among older participants. Given the dearth of comparative research across developmental periods, it is imperative to consider stage of development when examining the effects of CVE on mental health and the extent to which moderating factors may differentially support youth. The current study directly addresses this gap by testing school belonging as a potential buffer between CVE and mental health (depressive and PTSS) across two distinct developmental stages (early adolescence and young adulthood) captured in parallel school and university settings. Moreover, the inclusion of two developmentally distinct samples allows for the examination of whether school belonging operates similarly or serves different functions across early adolescence and young adulthood.

### School Belonging

School belonging, defined as the extent to which students feel accepted, respected, and supported within their school environment (CDC, 2024), is a central developmental resource in the context of adversity. Although the terms school belonging and school connectedness are often used interchangeably in the literature, important distinctions exist. School connectedness refers broadly to the extent to which students feel accepted, included, and cared for by staff and peers, while belonging more specifically captures students' emotional attachment to their school community (McCabe et al., 2024). Both constructs are typically conceptualized as components of broader school climate rather than distinct constructs (Bradshaw et al., 2014). In the present study, our measures are closely aligned with belonging, reflecting students' subjective sense of acceptance and inclusion. We retain both terms in line with prior literature while acknowledging this conceptual overlap.

Notably, the meaning and function of school belonging shift across developmental stages. In early adolescence, school environments often serve as primary sources of structure, safety, and adult support, making belonging particularly salient as youth navigate heightened sensitivity to social evaluation and identity formation (Wang & Eccles, 2012). For early adolescents, who are developmentally reliant on proximal adult protection, school belonging often functions as a protective container or surrogate family, offering consistent adult support, stable routines, and a sense of predictability that helps buffer the emotional consequences of CVE (Ozer & Weinstein, 2004; Wang & Eccles, 2012). Qualitative work revealed that adolescents in Mexico City constructed school belonging through informal peer relationships and mutual care, especially when formal school supports were limited (Saraví et al., 2020). Similarly, school belonging was a consistent predictor of psychological adjustment among Mexican adolescents, with feelings of support and inclusion linked to lower depressive symptoms (Sánchez et al., 2005).

In contrast, in young adulthood, school belonging reflects a broader sense of institutional fit, autonomy, and engagement, as students transition into more independent roles and future‐oriented pathways (Pittman & Richmond, 2007; Wilson & Gore, 2013). While not in the context of Mexico, prior research indicates that school belonging is linked to depressive symptoms among Latinx university students in the U.S. (Takimoto et al., 2021). In Mexico, school belonging has been qualitatively tied to greater academic engagement and persistence, as well as increased psychological well‐being among university students (Saraví et al., 2020). In a mixed methods study with university students in the Dominican Republic, affective bonds with peers emerged as the strongest predictor of institutional identification, suggesting that peer belonging remains central at the university level, though its function shifts from the safety‐seeking of early adolescence toward the formation of professional identity and long‐term social networks (Brea, 2014). Therefore, developmental differences suggest that school belonging may not operate uniformly across age groups. It is important to note that most research on school belonging has focused on K‐12 populations; comparatively far less is known about how it functions in university settings, especially within high‐violence contexts in Latin America. Thus, the present study addresses this gap by examining school belonging across two developmentally distinct groups (early adolescents in public middle schools and young adults in a public university) to assess whether its role differs in the context of CVE and mental health outcomes.

### Gender

Gender differences in both CVE exposure and psychological responses are well‐documented and theoretically relevant to the present study. With respect to exposure, boys consistently report higher rates of witnessing violence, personal victimization, and total CVE than girls, in part due to greater engagement in unstructured community‐based activities in high‐risk neighborhoods that increase their exposure to violence (Mora et al., 2022; Mora et al., 2023). In contrast, girls may be more affected by witnessing violence and its indirect psychological consequences, reflecting differences in the nature rather than simply the frequency of exposure (McDonald & Richmond, 2008). These differences in both the level and nature of exposure suggest that boys and girls may experience and respond to community violence in distinct ways, with implications for how CVE is linked to mental health outcomes.

Gender differences in psychological responses to CVE are similarly complex. Girls consistently report higher levels of depressive symptoms following violence exposure, whereas boys may bear a greater burden of PTSS under certain conditions (McDonald & Richmond, 2008; Miliauskas et al., 2022). Evidence from Mexican samples further highlights this variability. In a nationally representative sample of adolescents and young adults, females reported higher psychological distress than males across age groups (Pérez‐Sastré et al., 2024b). In contrast, among rural Mexican adult samples, men exposed to high levels of violence reported significantly greater PTSS than women, suggesting that gender differences in violence‐related outcomes depend on the nature of exposure and the developmental period examined (Leonard et al., 2025).

Taken together, these findings indicate that gender does not operate uniformly across outcomes or contexts. Rather, gender may shape both exposure to violence and psychological responses in ways that vary across developmental stages and types of CVE. Consistent with this perspective, prior work suggests that neighborhood violence and gender interact to influence adolescents' patterns of community engagement and vulnerability (Medrano et al., 2026). Accordingly, in the present study, gender is examined as a moderator of the associations between CVE and mental health outcomes. We further test whether gender operates as a higher order moderator by examining three‐way interactions between CVE, school belonging, and gender, allowing us to assess whether the role of school belonging differs for boys and girls across developmental stages, including early adolescence and young adulthood.

### Social Ecological Model of Resilience

This study draws on the Social Ecological Model of Resilience (SEMR) to examine whether school belonging moderates the associations between CVE and adolescent mental health outcomes, specifically depressive symptoms and PTSS. Within the SEMR, resilience is conceptualized as a dynamic, interactive process that unfolds within multiple systems of influence at the individual, relational, institutional, and community levels (Ungar, 2011). The model emphasizes that adaptive functioning in the face of adversity depends on youths' access to and engagement with meaningful, culturally relevant, and contextually available resources. As part of the microsystems of youth, particularly within contexts of chronic violence and instability, schools can provide protective relational and institutional assets that strengthen coping, belonging, and the development of core competencies such as emotion regulation, academic engagement, and problem‐solving. These competencies represent central pathways through which youth navigate community violence and demonstrate more positive psychological outcomes (Masten, 2014; Ungar & Theron, 2020). Importantly, the SEMR also highlights that the processes supporting resilience vary across developmental periods. During early adolescence, school‐based relationships and structures may scaffold emotional regulation, feelings of safety, and academic motivation, whereas during young adulthood, they may promote autonomy, identity coherence, and persistence in academic and community‐oriented goals (Eccles & Roeser, 2011; Pittman & Richmond, 2007). Applying the SEMR to this study therefore allows for a nuanced examination of how school‐based protective systems function across two distinct developmental stages, and how these systems may help youth maintain psychological well‐being amid pervasive violence.

### Study Context

The region of Michoacán provides a particularly compelling context for examining the effect of CVE and the role of school belonging as a protective factor across developmental stage. For over a decade, this region has consistently ranked among the most violent globally (Cure Violence Global, 2023; Seguridad, Justicia y Paz, 2022). In 2024, when the current studies were conducted, the site of the current study reported 55 homicides per 100,000 residents (Cure Violence Global, 2023), underscoring the pervasive and chronic threat of violence. In response to escalating cartel activity and violence in communities, the federal government deployed the National Guard and the Civil Guard in mid‐2024 to stabilize security conditions (Michoacan Government, n.d.). Although empirical research in this context itself remains scarce, findings from the nearby city of Morelia highlight regional patterns of poly‐victimization and daily exposure to danger among adolescents.

Méndez‐López and Pereda (2019) examined victimization rates among Mexican adolescents across different regions and found clear regional disparities in exposure rates. Adolescents from the states of Michoacán and Morelos reported the highest prevalence of both direct and indirect violence exposure, whereas those from northern Mexico showed comparatively lower rates. Specifically, poly‐victimization ranged from 16% in the state of Sonora to 31% in Michoacán, underscoring substantial geographic variation in the magnitude of violence exposure—likely reflecting differences in organized crime activity, community safety, and social cohesion across Mexican states (Méndez‐López & Pereda, 2019). Together, these conditions situate Michoacán as both an extreme case of violence and a microcosm of broader urban challenges in Mexico, where structural inequities and organized crime create persistent risks for youth.

### Present Study

The present study examines school belonging as a potential protective factor among early adolescents and young adults in Uruapan, Michoacán. Drawing on two developmentally distinct samples, this cross‐sectional study had five primary aims. First, we examined the prevalence of specific forms of community violence that early adolescents and young adults witness and are personally victimized by. Second, we examined developmental differences in the frequency and prevalence of exposure to community violence, and we expected that young adults would report greater exposure compared with early adolescents (H1). Third, we investigated how different forms of CVE, including witnessing violence and personal victimization, are associated with depressive and PTSS, hypothesizing that higher levels of exposure would be associated with greater symptoms (H2; Santacrose et al., 2021), and that personal victimization would show stronger associations than witnessing violence (H3; Mora et al., 2024). We also anticipated developmental differences in patterns of associations, with stronger links expected among early adolescents (H4; Russell et al., 2016). Fourth, we tested whether school belonging moderates associations between CVE and mental health outcomes, hypothesizing a moderating effect (H5). Finally, we explored whether this moderating effect varies by gender, testing a three‐way interaction to examine whether the role of school belonging differs for boys and girls.

## METHOD

### Participants and procedures

Data for the present study were collected in a large urban city in the state of Michoacán, Mexico. Prior to recruitment, the Principal Investigator (first author) met with middle school principals and the university director to describe the aims of the research and collaboratively finalize the themes to be included in each sample. These conversations ensured that survey content aligned with institutional priorities and reflected topics of particular relevance to the participating schools and university communities. All procedures and materials were reviewed and approved by the Institutional Review Board (IRB) at the University of Pittsburgh in the United States and by a collaborating IRB at a partner institution in Mexico (Servicios Educativos Integrados al Estado de Mexico [SEIEM: Integrated Educational Services of the State of Mexico]). Following IRB approvals, recruitment flyers were posted in high‐traffic areas (e.g., bathrooms, bulletin boards, hallways) across both sites. Given developmental and institutional differences, procedures varied slightly by sample and are described below.

### Early adolescent sample (MAPI study)

Participants were early adolescents recruited from public secondary schools in Uruapan, Michoacán, Mexico as part of the MAPI Study (Linking Neighborhood Violence to Mexican Adolescent Psychological and Immune Health Study). Eligibility criteria included: (a) enrollment in participating schools, (b) age between 10 and 17 years, and (c) parental consent and youth assent. The final sample included 346 adolescents (*M*_age_ = 13.02; SD = .98) from school A (*n* = 199; 57.5%) and school B (*n* = 147; 42.5%) from the sixth (*n* = 135; 39%), seventh (*n* = 109; 31.5%), and the eighth grade (*n* = 102; 29.5%). We refer to this sample as early adolescents in keeping with frameworks that define early adolescence as spanning approximately 10–14 years (Blum et al., 2014), consistent with the sample's mean age and predominantly sixth‐ through eighth‐grade composition. Students were roughly evenly distributed in gender, with 47.8% identifying as girls (*n* = 161) and 52.2% as boys (*n* = 176). An additional 2.6% identified as transwoman (*n* = 1), nonbinary (*n* = 2), or other (*n* = 6). Adolescents' paternal caregivers had varied educational attainment, with 6.4% reporting no formal schooling (*n* = 21), 55.2% reporting some elementary through completed middle school (*n* = 181), and 38.4% reporting some high school through completed secondary education (*n* = 126).

Monthly household income was assessed via adolescent self‐report and categorized into four groups. Among participants with available data (*n* = 112), income was generally low, with the largest proportions reporting ≤3000 MXN per month (*n* = 37; 33%) and between 3001 and 6000 MXN per month (*n* = 27; 24.1%). However, a substantial proportion of participants did not report income (67.6%), and given that this measure relied on adolescent report, it should be interpreted with caution. Accordingly, father's education was used as the primary indicator of socioeconomic status (SES) in analyses. Surveys were administered in Spanish via Qualtrics on tablets in group classroom settings during designated periods of the school day. Adolescents provided written parental consent and written assent prior to participation. Participants received 100 Mexican pesos (approximately USD $5.00) for survey completion.

### Young adult sample (Proyecto VENCER)

Participants were young adults recruited from the only local public university in the same region of Michoacán as part of Proyecto VENCER (Violence Exposure in College Mexican Emerging Adults and Resilience Study). Eligibility criteria included: (a) enrollment as a first‐ or second‐year student at the university, and (b) age 18 or older. The analytic sample included 499 participants (*M*_age_ = 19.39; SD = 2.55) who were in their first (*n* = 292; 58.5%) or second year (*n* = 204; 40.9%). Most students identified as women (63.1%; *n* = 315), followed by 35.5% who identified as men (*n* = 177). In terms of schooling, 47.6% (*n* = 222) of participants' fathers had no formal education or had completed schooling through middle school, while 52.4% (*n* = 244) had completed at least some high school through a professional degree. Following the same coding procedure for early adolescents, monthly household income was assessed via young adults' self‐report and categorized into four groups. Among participants with available data (*n* = 335), income was skewed toward the higher categories, with the largest proportion reporting monthly household incomes greater than 10,000 MXN (*n* = 178; 53.1%), followed by those reporting between 6001 and 10,000 MXN (*n* = 84; 25.1%). Approximately, one‐third of participants did not report income (32.9%), and given that this measure relied on self‐report, it should be interpreted with caution. Accordingly, father's education was used as the primary indicator of SES in analyses.

Surveys were administered in Spanish using paper‐and‐pencil questionnaires. A team of trained research assistants independently entered responses into Qualtrics and cross‐checked entries to ensure accuracy. Participants provided written informed consent prior to participation and received 85 Mexican pesos (approximately USD $4.50) for survey completion. We deliberately use the term *young adults* rather than *emerging adults* to reflect the sociodemographic context of the region. In settings marked by structural violence and economic precarity, transitions to adulthood are often accelerated, limiting the extended period of identity exploration typically associated with emerging adulthood in WEIRD contexts (Arnett, 2016; Galambos & Martínez, 2007).

### Measures

#### Community violence exposure (CVE)

Community violence exposure was assessed using the Survey of Exposure to Community Violence (Richters & Martinez, 1993), expanded for urban contexts by Brennan et al. (2007). This measure has been reliably used in Spanish with Mexican adolescent and adult samples (Kabir et al., 2025; Mora et al., 2024). Participants reported how often they witnessed or directly experienced violent events in their neighborhoods, excluding anything seen in the media. The measure includes two subscales: witnessing violence (13 items; e.g., “Have you seen someone else get chased by gangs or individuals when you thought they could get really hurt?”), and personal victimization (9 items; e.g., “Have you, yourself, been threatened with serious physical harm by someone?”). Items were rated on a Likert scale from 0 (*Never*) to 7 (*Seven or more times*). Items within each subscale were sum scored, with higher scores indicating greater exposure in the past year. Possible scores ranged from 0 to 84 for witnessing violence and 0–70 for personal victimization. Witnessing violence and personal victimization were examined as separate indicators of community violence. Cronbach's alphas were acceptable in each sample (early adolescents: = .85 witnessing violence; *α* = .88 personal victimization; young adults: *α* = .87 witnessing violence; *α* = .72 personal victimization).

#### School belonging

School belonging for early adolescents was assessed with five items that capture students' feelings of acceptance, inclusion, and safety within their school environment (e.g., I feel close to people in my school; McNeely et al., 2002). This scale is appropriate for adolescents and has been reliably used with a sample of Mexican adolescents (Boyce et al., 2023). Students responded on a Likert scale from 1 (*Very strongly disagree*) to 5 (*Very strongly agree*). Consistent with recommendations for handling item‐level missingness (Newman, 2014), school belonging was computed as the mean of five items for participants with at least three responses. Cronbach's alpha was .79.

School belonging for young adults was assessed using the School Belonging Experiences subscale from Brea's (2014) Institutional Sense of Belonging measure. The subscale includes 20 items assessing the extent to which students experience belonging‐related features of their university environment (e.g., “You are proud to belong to this University”). Participants responded to the prompt, “How much do you experience this at your university?” using a 4‐point Likert scale ranging from 1 (*Very little*) to 4 (*A lot*). Items were mean‐scored to create a composite index of school belonging (possible range 1–4), with higher scores reflecting greater perceived school belonging and engagement with institutional life. This scale is appropriate for Latine samples and has been reliably used with a sample of Latin American university students (Aramendiz González & Delgadillo Correa, 2021). Cronbach's alpha was .89.

#### Depressive symptoms

Depressive symptoms were assessed using the Patient Health Questionnaire (PHQ‐9), a 9‐item self‐report scale developed by Spitzer (1999) to measure the frequency of symptoms in the past 2 weeks. A sample item includes, “Little interest or pleasure in doing things.” Participants responded on a 4‐point scale from 0 (*Not at all*) to 3 (*Nearly every day*). Items were summed to create a total depressive symptom score (range = 0–27), with higher scores indicating greater symptom severity. The PHQ‐9 has demonstrated strong reliability and validity in adolescent and young adult populations, including Spanish‐speaking samples (Errazuriz et al., 2023; Richardson et al., 2010). Cronbach's alpha was .91 in both samples.

#### Posttraumatic stress symptoms (PTSS)

Posttraumatic stress symptoms (PTSS) were assessed using the Posttraumatic Stress Disorder Checklist–Civilian Version (PCL‐C), a 17‐item self‐report measure designed to evaluate PTSS in civilian populations (Weathers et al., 1993). Items align with DSM‐IV diagnostic criteria and assess symptoms experienced in the past month related to a stressful or traumatic event, including avoidance, emotional numbing, hyperarousal, and intrusive memories. A sample item includes, “Repeated, disturbing memories, thoughts, or images of a stressful experience from the past.” Responses were rated on a 5‐point scale from 1 (*Not at all*) to 5 (*Extremely*). Items were summed to create a total PTSS severity score (possible range = 17–85), with higher scores reflecting greater symptom severity. The PCL‐C has been validated in Spanish (Miles et al., 2008) and has been reliably used with Mexican adults and adolescents (Mora et al., 2024). Cronbach's alpha was .94 for both early adolescents and young adults.

#### Covariates

Adolescent demographics (age, gender, employment status, and father's highest level of education) were included as covariates, as prior research has revealed associations with violence exposure and psychological outcomes (Leonard et al., 2025). Gender was coded as 0 for adolescent girl and 1 for adolescent boy, and school site was coded as 0 = School A, 1 = School B. Father's highest level of education was used as a proxy for SES and was coded on an ordinal scale ranging from 0 (*No formal schooling*) to 9 (*Professional degree*).

## ANALYTIC PLAN

Data cleaning and analysis were conducted in SPSS V28.1. Preliminary descriptive statistics and tests of normality were examined separately for the early adolescent and young adult samples. To address Aim 1, frequencies and descriptive statistics for witnessing violence and personal victimization were calculated within each developmental group. For Aim 2, exposure to community violence was examined descriptively within each sample. We report mean frequency scores for each item and subscale, and dichotomized indicators were used to estimate the prevalence of exposure. Given the descriptive aims of this analysis and the nonnormal distribution of the item‐level frequency scores, we did not conduct formal statistical comparisons of exposure across groups. Given that the current study is based on nonprobability, school‐based samples, findings should be interpreted as reflecting sample‐based estimates rather than population‐level prevalence. To address Aim 3, we examined associations between CVE and mental health outcomes (i.e., depressive symptoms and PTSS) using hierarchical regression models conducted separately for early adolescents and young adults. For each sample, depressive symptoms and PTSS were modeled in separate regressions. Given their high correlation, witnessing violence and personal victimization were examined in separate models.

To address Aim 4, moderation by school belonging was tested using parallel hierarchical regression models following Aiken et al. (1991) protocols. For each sample, in Step 1, demographic covariates were entered. For early adolescents, covariates included age, father's highest level of education, and school site. However, results were robust to the inclusion of school site as a covariate, with no meaningful changes in the pattern or significance of findings; therefore, for parsimony and comparability across developmental groups, final models are presented without school site. For young adults, covariates included age, employment status, and father's highest level of education. In Step 2, the mean‐centered independent variable (CVE) and moderator (school belonging) were entered. In Step 3, all two‐way interaction terms (violence × school belonging, violence × gender, and school belonging × gender) were entered. In Step 4, the three‐way interaction (violence × school belonging × gender) was included. Significant interactions were probed using simple slopes analyses at ±1 SD of the moderator(s) using the PROCESS macro (Hayes, 2012). Given differences in sample size and variability across developmental groups, comparisons of statistical significance should be interpreted cautiously; therefore, emphasis is placed on the magnitude and patterning of effects across samples.

## RESULTS

### Preliminary results

As shown in Table 1, exposure to community violence was widespread among both early adolescents and young adults in Uruapan. Nearly all participants had heard gunfire near their homes (82.3% of adolescents; 94.6% of young adults) and a majority had witnessed other violent events, such as seeing someone get hit, slapped, or punched (59.5% of adolescents; 57.9% of young adults) or using or selling illegal drugs (56.5% of adolescents; 72.9% of young adults). Personal victimization was also common, with 79.9% of adolescents and 68.5% of young adults reporting at least one direct experience of violence, most often being hit, slapped, or punched. Descriptively, a higher proportion of young adults than early adolescents endorsed witnessing gunfire, weapon carrying, and drug activity, whereas a higher proportion of early adolescents endorsed direct victimization, including being beaten up, attacked with a weapon, and shot at. Overall, both groups faced high and chronic levels of direct and indirect violence exposure in their communities. Taken together, these findings indicate that exposure to community violence is not only highly prevalent but also spans multiple forms of violence, consistent with patterns of poly‐victimization observed in similar high‐risk contexts.

**TABLE 1:** Frequency of violence exposure among early adolescents and young adults by type of witnessing and personal victimization.

Violence item	Study 1: Early adolescents		Study 2: Young adults	
	*M* (SD)	%	*M* (SD)	%
**Witnessing violence**	15.55 (14.14)	89.4	18.78 (14.35)	98.6
Heard gunfire outside or near your home	3.41 (2.67)	82.3	4.59 (2.40)	94.6
Seen other people using or selling illegal drugs	1.86 (2.41)	56.5	0.27 (2.59)	72.9
Seen someone get hit, slapped, or punched by someone	1.77 (2.27)	59.5	1.57 (1.93)	57.9
Seen someone carrying or holding a gun or knife	1.73 (2.23)	58.3	2.29 (2.33)	70.6
Heard gunfire outside in or near your school building	1.44 (2.08)	52.8	1.24 (1.89)	47.5
Seen someone being threatened with serious physical harm	1.20 (1.80)	51.1	1.41 (1.94)	51.5
Seen a dead person somewhere in the community	1.06 (1.65)	45.3	1.26 (1.79)	51.4
Seen someone getting beaten up or mugged	1.06 (1.76)	42.4	0.79 (1.41)	38.0
Seen a seriously wounded person after an incident of violence	0.92 (1.70)	40.2	0.95 (1.42)	47.9
Seen someone get shot at, but not wounded, with a gun	0.55 (1.30)	24.7	0.36 (0.88)	20.9
Seen someone being attacked or stabbed with a weapon	0.51 (1.29)	21.7	0.33 (0.94)	17.6
Seen someone get shot and wounded with a gun	0.50 (1.25)	22.6	0.54 (1.20)	26.2
Seen someone get chased by gangs or individuals	0.12 (0.68)	28.3	0.79 (1.42)	37.1
**Personal victimization**	5.65 (9.37)	79.9	3.90 (5.60)	68.5
Hit, slapped, or punched by someone	1.29 (2.13)	42.8	0.65 (1.45)	27.1
Directly threatened with serious physical harm by someone	0.86 (1.60)	34.6	0.81 (1.61)	32.5
Chased by gangs or individuals	0.72 (1.52)	28.3	0.46 (0.98)	26.5
At home when someone has broken into your home	0.60 (1.35)	26.9	0.38 (0.86)	23.8
Asked to get involved in selling or distributing illegal drugs	0.59 (1.36)	23.2	0.71 (1.72)	22.4
Beaten up or mugged	0.58 (1.48)	19.4	0.21 (0.63)	13.5
Attacked with a weapon like a bat or a knife (not a gun)	0.41 (1.29)	13.9	0.09 (0.50)	5.4
Shot at, but not wounded, with a gun	0.41 (1.37)	11.7	0.08 (0.44)	5.2
Shot at with a gun	0.33 (1.13)	10.5	0.01 (1.27)	1.0

*Note*: Values represent mean frequency scores (*M*, SD) and percentage of participants endorsing each type of violence exposure (1 = Has experienced this type of violence at least once). All values are presented descriptively. Bolded categories represent composite domain totals (Witnessing violence, Personal victimization).

Bivariate correlations among study variables revealed several notable patterns (see Tables 2 and 3). As expected, depressive symptoms and PTSS were strongly associated in both samples (*r*s = .65–.67, *p*s < .001). Greater exposure to witnessing and personal victimization showed moderate, positive associations with mental health symptoms across studies (*r*s = .21–.29, *p*s < .001). Violence exposure indicators were also strongly correlated with one another, particularly witnessing and personal victimization (*r*s = .66–.85, *p*s < .001). Lastly, school belonging showed small, inverse associations with depressive symptoms in the young adult sample (*r* = −.12, *p* < .01) but was otherwise unrelated to mental health indicators. Results are presented separately for each age group to characterize patterns within each developmental sample.

**TABLE 2:** Correlations between study variables for early adolescents.

Variable	1	2	3	4	5	6	7	8
1. Age	—							
2. Gender^a^	.05	—						
3. Employment status^b^	.15***	−.05	—					
4. Father's education^c^	.14**	.15**	.01	—				
5. PTSS	−.03	−.12**	−.11*	−.06	—			
6. Depressive symptoms	−.06	−.18***	−.14**	−.08	.67***	—		
7. Witnessing violence	.05	.14**	.13**	−.09*	.29***	.21***	—	
8. Personal victimization	.04	.14**	.17***	−.09	.28***	.19***	.66***	—
9. School belonging	.10*	.04	.07	.06	−.09	−.12**	.06	.00

*Note*: *p < .05, **p < .01, ***p < .001.

Abbreviation: PTSS, posttraumatic stress symptoms.

^a^ Gender was coded as 0 = girl and 1 = boy.

^b^ Employment status was coded as 0 = does not work and 1 = works.

^c^ Socioeconomic status (SES) was assessed using the father's highest level of education, coded from 0 = no formal schooling to 9 = professional degree.

**TABLE 3:** Correlations between study variables for young adults.

Variable	1	2	3	4	5	6	7	8
1. Age	—							
2. Gender^a^	−.01	—						
3. Employment status^b^	.13*	.21***	—					
4. Father's education^c^	.12*	.05	−.03	—				
5. PTSS	−.05	−.13*	.09	−.03	—			
6. Depressive symptoms	.03	−.23***	−.04	.05	.65***	—		
7. Witnessing violence	.06	.10	.17**	−.03	.23***	.38***	—	
8. Personal victimization	.06	.09	.20***	−.01	.25***	.43***	.85***	—
9. School belonging	.09	−.02	−.06	.06	.12*	.02	−.01	−.11*

*Note*: *p < .05, **p < .01, ***p < .001.

Abbreviation: PTSS, posttraumatic stress symptoms.

^a^ Gender was coded as 0 = woman and 1 = man.

^b^ Employment status was coded as 0 = does not work and 1 = works.

^c^ Socioeconomic status (SES) was assessed using the father's highest level of education, coded from 0 = no formal schooling to 9 = professional degree.

### Early adolescent sample

Hierarchical regression models examined associations between CVE (witnessing violence and personal victimization), school belonging, gender, and depressive symptoms and PTSS. None of the demographic covariates were significantly associated with depressive symptoms or PTSS (all *p*s > .05; Tables 4 and 5). Across outcomes, both witnessing violence and personal victimization were statistically significantly and positively associated with symptoms (all *p*s < .001), consistent with hypotheses that greater exposure would be linked to greater psychological symptoms (H2). Girls reported higher levels of depressive and PTSS symptoms than boys (Tables 4 and 5). School belonging was also positively associated with both depressive and PTSS symptoms (*p*s < .05; Tables 4 and 5).

**TABLE 4:** Hierarchical regression examining associations between community violence exposure and depressive symptoms.

	Early adolescents			Young adults		
	B	SE	*β*	B	SE	*β*
**Witnessing violence model**						
Step 1						
(Constant)	4.70	5.78		15.82***	2.46	
Age	0.43	0.44	0.06	−0.19	0.13	−0.07
Employment status^a^	−0.05	1.06	−0.002	−1.84**	**0.66**	**−0.13**
Father's education^b^	−0.01	0.16	−0.003	−0.03	0.02	−0.07
	*F* = 0.31; *R* ^2^ = .01			*F* = 4.67**; *R* ^2^ = .03		
Step 2						
Gender^c^	−3.62***	**0.79**	**−0.25**	2.14***	**0.66**	**0.15**
Witnessing violence	0.16***	**0.03**	**0.31**	0.13***	**0.02**	**0.27**
School belonging	0.18*	**0.08**	**0.17**	−1.22*	**0.57**	**−0.10**
	*F* = 6.76***; *R* ^2^ = .18; Δ*R* ^2^ = .16			*F* = 10.17***; *R* ^2^ = .12; Δ*R* ^2^ = .11		
Step 3						
Witnessing violence × School belonging	0.01	0.01	0.10	−0.03	0.04	−0.03
Witnessing violence × Gender	−0.07	0.06	−0.10	−0.02	0.05	−0.02
School belonging × Gender	0.02	0.17	0.01	1.41	1.21	0.09
	*F* = 9.37***; *R* ^2^ = .18; Δ*R* ^2^ = .16			*F* = 7.04***; *R* ^2^ = .13; Δ*R* ^2^ = .11		
Step 4						
Witnessing × Belong × Gender	−0.02	0.01	−0.10	0.05	0.07	0.04
	*F* = 6.28***; *R* ^2^ = .18; Δ*R* ^2^ = .16			*F* = 6.38***; *R* ^2^ = .13; Δ*R* ^2^ = .10		
**Personal victimization model**						
Step 1						
(Constant)	4.52	5.79		15.82***	2.46	
Age	0.45	0.44	0.06	−0.19	0.13	−0.07
Employment status^a^	−0.08	1.06	−0.04	−1.84**	0.66	−0.13
Father's education^b^	−0.03	0.16	−0.01	−0.03	0.02	−0.07
	*F* = 0.35***; *R* ^2^ = −.007			*F* = 4.67**; *R* ^2^ = .03		
Step 2						
Gender^c^	−3.84***	**0.81**	**−0.26**	2.09**	**0.66**	**0.15**
Personal victimization	0.21***	**0.05**	**0.26**	0.34***	**0.06**	**0.27**
School belonging	0.20*	**0.09**	**0.13**	−0.96	0.57	−0.08
	*F* = 7.48***; *R* ^2^ = .13; Δ*R* ^2^ = .12			*F* = 10.07***; *R* ^2^ = .12; Δ*R* ^2^ = .11		
Step 3						
Personal victimization × School belonging	0.02*	**0.01**	**0.14**	−0.07	0.10	−0.03
Personal victimization × Gender	−0.18	0.10	−0.18	0.05	0.12	0.02
School belonging × Gender	0.03	0.17	0.01	1.30	1.22	0.08
	*F* = 5.88***; *R* ^2^ = .16; Δ*R* ^2^ = .13			*F* = 6.94***; *R* ^2^ = .13; Δ*R* ^2^ = .11		
Step 4						
Victimization × Belonging × Gender	−0.06**	**0.02**	**−0.25**	−0.001	0.20	0.00
	*F* = 6.45***; *R* ^2^ = .18; Δ*R* ^2^ = .16			*F* = 6.23***; *R* ^2^ = .13; Δ*R* ^2^ = .11		

*Note*: Separate regression models are presented for witnessing violence and personal victimization. Continuous predictors were mean‐centered prior to analysis. *p < .05, **p < .01, ***p < .001. Bold values indicate statistically significant estimates, consistent with the significance levels denoted by asterisks.

^a^ Employment status was coded as 0 = does not work and 1 = works.

^b^ Highest level of education was used as a proxy for socioeconomic status, coded from 0 = no formal schooling to 9 = professional degree.

^c^ Gender was coded as 0 = girl/woman and 1 = boy/man.

**TABLE 5:** Hierarchical regression examining associations between community violence exposure and posttraumatic stress symptoms.

	Early adolescents			Young adults		
	B	SE	*β*	B	SE	*β*
**Model 1**						
Step 1						
(Constant)	48.05***	12.84		29.52***	5.42	
Age	−0.66	0.98	−0.04	−0.26	0.28	−0.05
Employment status^a^	3.28	2.35	0.08	−3.07*	**1.45**	**−0.10**
Father's education^b^	−0.21	0.36	−0.03	−0.05	0.05	−0.05
	*F* = 0.90; *R* ^2^ = .01			*F* = 2.36; *R* ^2^ = .02		
Step 2						
Gender^c^	−6.44***	**1.74**	**−0.20**	3.38*	**1.42**	**0.11**
Witnessing violence	0.44***	**0.06**	**0.37**	0.36***	**0.05**	**0.33**
School belonging	0.37*	**0.18**	**0.11**	−2.95*	**1.24**	**−0.11**
	*F* = 11.21***; *R* ^2^ = .19; Δ*R* ^2^ = .17			*F* = 11.70***; *R* ^2^ = .14; Δ*R* ^2^ = .13		
Step 3						
Witnessing violence × School belonging	0.01	0.01	0.04	−0.22**	**0.08**	**−0.13**
Witnessing violence × Gender	−0.01	0.13	−0.003	0.13	0.10	0.09
School belonging × Gender	0.20	0.37	0.04	−0.22	0.08	−0.13
	*F* = 7.50***; *R* ^2^ = .19; Δ*R* ^2^ = .16			*F* = 9.15***; *R* ^2^ = .16; Δ*R* ^2^ = .14		
Step 4						
Witnessing × Belonging × Gender	−0.01	0.03	−0.03	0.23	0.16	0.09
	*F* = 6.74***; *R* ^2^ = .19; Δ*R* ^2^ = .16			*F* = 8.47***; *R* ^2^ = .16; Δ*R* ^2^ = .14		
**Model 2**						
Step 1						
(Constant)	47.86***	12.89		29.52***	5.42	
Age	−0.64	0.99	−0.04	−0.26	0.28	−0.05
Employment status^a^	3.23	2.36	0.08	−3.07*	1.45	−0.10
Father's education^b^	−0.23	0.36	−0.04			
	*F* = 0.98; *R* ^2^ = .01			*F* = 2.36; *R* ^2^ = .02		
Step 2						
Gender^c^	−7.27***	**1.76**	**−0.22**	3.35*	**1.41**	**0.11**
Personal victimization	0.67***	**0.10**	**0.37**	0.99***	**0.13**	**0.36**
School belonging	0.47*	**0.18**	**0.14**	−2.23	1.23	−0.08
	*F* = 10.99***; *R* ^2^ = .18; Δ*R* ^2^ = .17			*F* = 12.89***; *R* ^2^ = .15; Δ*R* ^2^ = .14		
Step 3						
Personal victimization × School belonging	0.04	0.02	0.10	−0.48*	**0.20**	**−0.11**
Personal victimization × Gender	−0.34	0.22	−0.16	0.36	0.25	0.09
School belonging × Gender	0.27	0.36	0.06	0.95	2.61	0.03
	*F* = 7.97***; *R* ^2^ = .20; Δ*R* ^2^ = .17			*F* = 9.53***; *R* ^2^ = .17; Δ*R* ^2^ = .15		
Step 4						
Victimization × Belonging × Gender	−0.06	0.04	−0.10	0.32	0.42	0.04
	*F* = 7.36***; *R* ^2^ = .20; Δ*R* ^2^ = .18			*F* = 8.63***; *R* ^2^ = .17; Δ*R* ^2^ = .15		

*Note*: Separate regression models are presented for witnessing violence and personal victimization. Continuous predictors were mean‐centered prior to analysis. *p < .05, **p < .01, ***p < .001. Bold values indicate statistically significant estimates, consistent with the significance levels denoted by asterisks.

^a^ Employment status was coded as 0 = does not work and 1 = works.

^b^ Socioeconomic status (SES) was assessed using the father's highest level of education, coded from 0 = no formal schooling to 9 = professional degree.

^c^ Gender was coded as 0 = girl/woman and 1 = boy/man.

**FIGURE 1:**
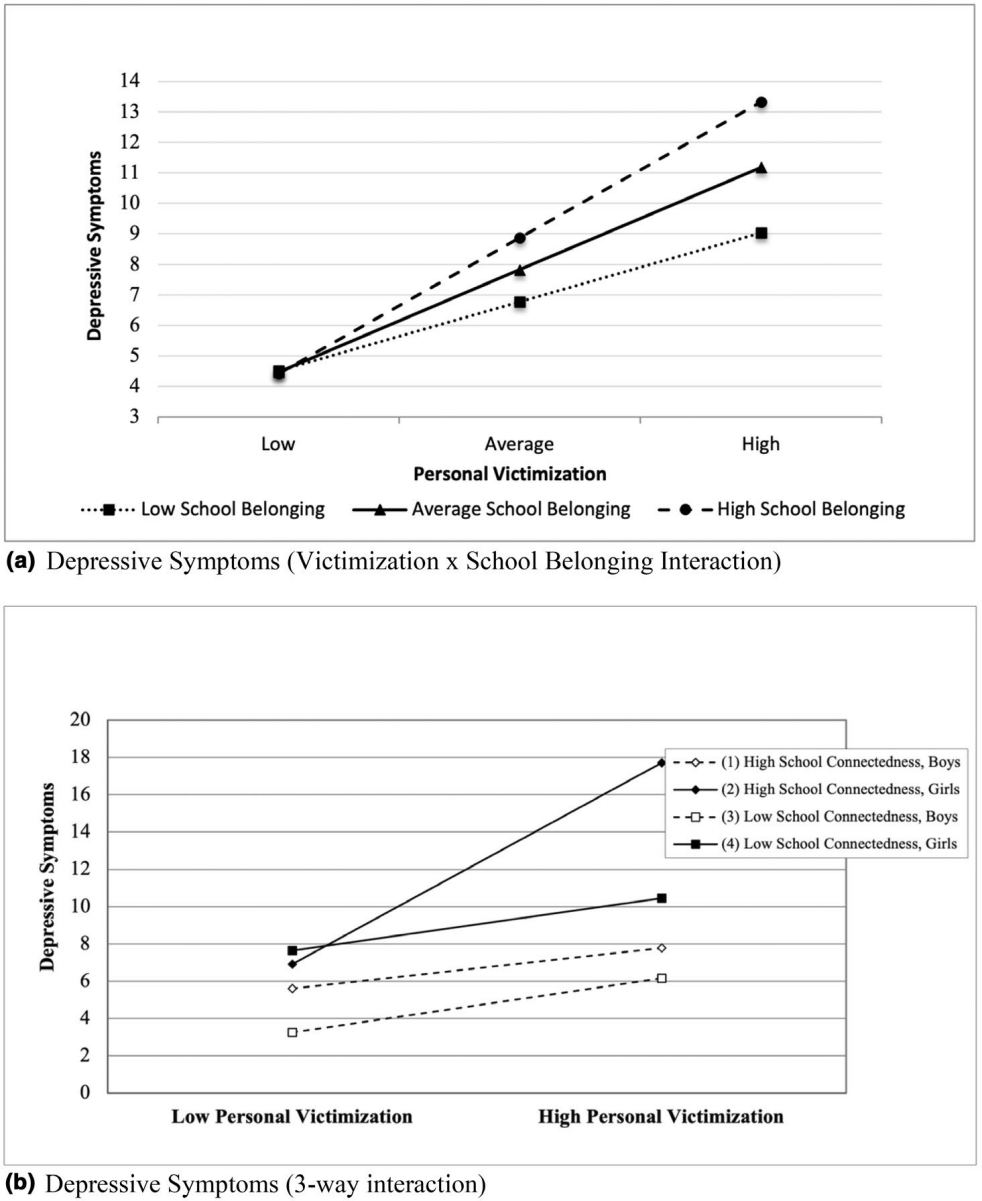
School belonging as a moderator of associations between personal victimization and mental health among early adolescents. Panel A depicts associations among personal victimization, school belonging, and gender in relation to depressive symptoms. Panel B depicts associations between personal victimization and PTSS as a function of school belonging. Values represent predicted symptom levels at low (−1 SD) and high (+1 SD) levels of school belonging.

For depressive symptoms, the inclusion of primary correlates accounted for a meaningful proportion of variance (*R* ^2^ = .133), with two‐way interactions explaining additional variance (Δ*R* ^2^ = .022) and the three‐way interaction contributing further incremental variance (Δ*R* ^2^ = .028; final *R* ^2^ = .183; Table 4). A significant interaction between personal victimization and school belonging emerged and was further moderated by a three‐way interaction with gender (Table 4), supporting our hypothesis that school belonging would moderate associations between CVE and mental health (H5), though not in the expected direction. Probing of this interaction (see Figure 1, Panels A,B) indicated that the association between victimization and depressive symptoms strengthened with higher school belonging among girls but attenuated and became nonsignificant among boys, suggesting a gender‐differentiated pattern of moderation. For PTSS, primary correlates similarly accounted for variance in symptoms (*R* ^2^ = .183), with interaction terms contributing relatively small incremental variance (Δ*R* ^2^ = .015 for two‐way interactions; Δ*R* ^2^ = .004 for the three‐way interaction; final *R* ^2^ = .202; Table 5). No interaction effects were observed for witnessing violence or for PTSS (Table 5), failing to support our hypothesis that school belonging would moderate associations between violence exposure and PTSS (H5). These findings suggest that associations with PTSS were relatively consistent across levels of school belonging and gender (see Figure 1, Panel A).

### Young adult sample

Across models, employment status was consistently associated with lower depressive and PTSS symptoms (*p*s ≤ .035), whereas age and father's education were not significantly associated with outcomes (Tables 4 and 5). Violence exposure was statistically significantly and positively associated with depressive symptoms across both models, with higher levels of witnessing violence and personal victimization linked to greater depressive symptoms (*p*s < .001; Table 4), consistent with our hypothesis (H2). Additionally, women reported higher levels of depressive symptoms than men (*p*s ≤ .023; Table 4). School belonging was not significantly associated with depressive symptoms in models including personal victimization but demonstrated a small negative association in models including witnessing violence (*p* ≤ .05; Table 4), such that greater belonging was linked to lower depressive symptoms.

**FIGURE 2:**
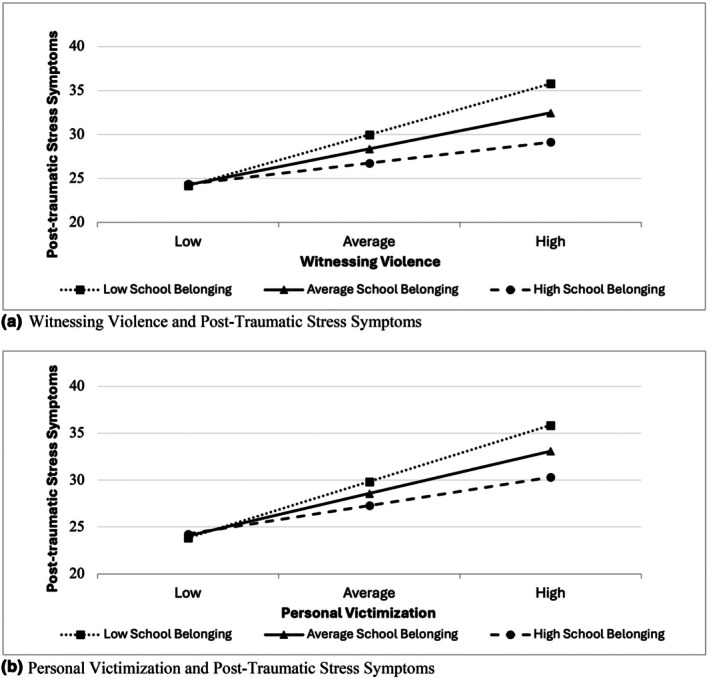
School belonging as a moderator of associations between violence exposure and posttraumatic stress symptoms among young adults. Panel A depicts associations between witnessing violence and posttraumatic stress symptoms across levels of school belonging. Panel B depicts associations between personal victimization and PTSS across levels of school belonging. Values represent predicted posttraumatic stress symptoms at low (−1 SD), mean, and high (+1 SD) levels of school belonging.

For depressive symptoms, primary correlates accounted for a modest proportion of variance (*R* ^2^ = .122), with interaction terms contributing minimal additional variance (Δ*R* ^2^≈.004; final *R* ^2^ = .126; Table 4). No significant interaction effects emerged between CVE and school belonging, and there was no evidence of gender‐based moderation (all *p*s > .05; Table 4), failing to support hypotheses that school belonging would moderate associations between violence exposure and depressive symptoms (H5). Associations between violence exposure and depressive symptoms were therefore largely consistent across levels of school belonging and gender (see Figure 2, Panel A). Violence exposure was also statistically significantly positively associated with PTSS symptoms (*p*s < .001), and women reported higher levels of PTSS symptoms than men (*p*s ≤ .023; Table 5), again consistent with Hypothesis 2. School belonging demonstrated a negative association with PTSS symptoms in models involving witnessing violence (*p*s ≤ .032; Table 5) and was not significantly associated in personal victimization models. For PTSS, primary correlates accounted for a meaningful proportion of variance (*R* ^2^ = .150), with interaction terms contributing a small but nontrivial increment (Δ*R* ^2^≈.016; final *R* ^2^ = .166; Table 5). Moderation effects emerged for PTSS, such that associations between both witnessing violence and personal victimization and PTSS varied as a function of school belonging (*p*s ≤ .018; Table 5), supporting our hypothesis that school belonging would moderate associations between CVE and mental health (H5). Simple slopes analyses (−1 SD and +1 SD; see Figure 2, Panel B) indicated that associations between violence exposure and PTSS were stronger at lower levels of school belonging and attenuated at higher levels, consistent with a buffering pattern. No evidence of gender‐based moderation emerged for PTSS (all *p*s > .05; Table 5), failing to support hypotheses regarding gender‐based moderation.

## DISCUSSION

The present study examined how CVE relates to depressive symptoms and PTSS across early adolescence and young adulthood, and whether school belonging serves as a protective factor in these associations. Understanding these patterns is essential in regions marked by chronic violence because adolescents and young adults must navigate major developmental transitions in environments that threaten safety, belonging, and psychological outcomes. Descriptively, we found that a substantial proportion of early adolescents and young adults reported witnessing or personal victimization in their communities, which is consistent with patterns documented in regions affected by organized crime and neighborhood instability (Méndez‐López et al., 2021). These descriptive findings highlight the pervasiveness and breadth of CVE across both developmental groups. Importantly, exposure was not uniform across types of violence, with some forms (e.g., hearing gunfire, witnessing drug activity, and observing weapons) occurring far more frequently than others. High levels of both witnessing violence and personal victimization further suggest that exposure in this context is not limited to isolated events, but rather reflects engagement across multiple forms of violence, consistent with a poly‐victimization framework (Finkelhor et al., 2007).

Although exposure was widespread for both groups, the patterns differed by type of violence. Young adults reported higher levels of witnessing violence across most items, with 98.6% reporting at least one witnessed violent event compared with 89.4% of adolescents. Young adults were also more likely to report seeing weapons, hearing gunfire near their homes, and observing drug use or sales. In contrast, early adolescents (79.7% vs. 68.5%) reported higher levels of personal victimization. Specifically, adolescents were more likely to experience direct physical harm such as being hit or punched, being chased, having their homes broken into while present, and being attacked with a weapon. These patterns suggest that early adolescents face greater direct interpersonal threat, while young adults are more frequently exposed to witnessing violent events occurring around them.

The differences in witnessing and personal victimization across age groups likely reflect developmental and contextual factors that shape daily routines and exposure opportunities. Early adolescents typically spend more time in their immediate neighborhoods, engage in more peer interactions close to home, and have limited autonomy in deciding where and when they move through their communities (Kim et al., 2018). These constraints increase the likelihood of direct interpersonal encounters, which may explain their higher rates of personal victimization. Young adults often have greater mobility, spend more time in public or community spaces, and navigate environments where violent events may be more visible (Scarpa, 2003). This broader movement increases the likelihood of witnessing violence, even if they are less frequently the direct targets of aggression. These developmental differences indicate that age not only influences the type of violence youth encounter but also the social contexts in which exposure occurs. Understanding these patterns is important because witnessing and personal victimization may place youth at different types of risk and contribute to psychological outcomes through distinct mechanisms.

Each form of exposure represents a different type of chronic stress that may shape psychological outcomes in distinct ways. One possible interpretation is that early adolescents who feel deeply connected to school may experience a sharper emotional violation when community conditions contradict the safety and predictability they associate with the school microsystem (Gaias et al., 2018). Within the early adolescent sample, the contrast between a supportive school environment and the dangers present in the broader community may heighten stress reactivity, particularly during a developmental stage marked by heightened socioemotional sensitivity and reliance on proximal adults (Steinberg, 2014). This account may help explain why greater school belonging was linked to a stronger, rather than weaker, association between CVE and depressive symptoms among early adolescents. We offer it tentatively, however, because our measure asked about violence in the neighborhood rather than at school, with the single exception of one item on hearing gunfire near school, and we did not test whether the location of exposure shaped these associations. We therefore cannot fully rule out that some exposure occurred near school settings, which would complicate a strict distinction between a safe school and a dangerous community.

Across both early adolescence and young adulthood, CVE, whether through witnessing violence or personal victimization, was consistently associated with higher levels of depressive symptoms and PTSS. These findings underscore the pervasive impact of CVE on mental health and align with prior research in Mexican samples documenting similar associations (Mora et al., 2024; Pérez‐Sastré et al., 2024b). Aligned with prior research (e.g., Leonard et al., 2025), personal victimization showed the strongest associations with psychological distress, indicating that being personally targeted by violence may be particularly harmful for emotional well‐being. Witnessing violence was also associated with poorer mental health, reflecting the emotional burden of indirect exposure and supporting the idea that both forms of CVE meaningfully contribute to depressive symptoms and PTSS. Hierarchical regression analyses further clarified these associations. After accounting for demographic factors, both witnessing violence and personal victimization remained significantly associated with depressive symptoms and PTSS across developmental stages. Among the early adolescents, personal victimization exerted especially strong effects on both outcomes, highlighting younger adolescents' heightened sensitivity to direct violence (Herrenkohl et al., 2012). Witnessing violence also contributed to symptoms, though to a lesser extent, consistent with the role of vicarious stress and heightened threat perception (Fowler et al., 2009).

The present findings also revealed that school belonging interacts with CVE in complex, developmentally dependent ways. Importantly, because school belonging was measured using different instruments across the two samples and reflects a developmentally distinct construct in each context, the present study is not positioned to establish whether the role of belonging statistically differs across developmental groups. Rather, findings are characterized in terms of how belonging operated within each sample separately, and patterns across samples are noted as descriptive observations rather than formal comparative conclusions. In early adolescence, school belonging appeared to intensify the association between personal victimization and depressive symptoms, consistent with a vulnerable‐reactive effect (Luthar et al., 2000). Adolescents who were strongly connected to school may have experienced greater emotional distress when their expectations of safety and support stood in contrast to their exposure to community violence. This pattern suggests that school belonging does not uniformly buffer the links between violence and distress during early adolescence and may, under certain circumstances, be associated with greater distress.

In contrast, school belonging began to show protective effects for PTSS among young adults, suggesting that supportive academic environments may buffer the effect of community violence as youth gain greater autonomy and coping resources. This pattern aligns with prior research in university contexts demonstrating that greater school belonging is associated with lower psychological distress and can buffer the effects of stress exposure (e.g., Pittman & Richmond, 2007; Wilson & Gore, 2013). The specificity of the buffering effect for PTSS, but not depressive symptoms, suggests that school belonging in young adulthood may operate primarily through mechanisms tied to threat reduction, such as perceived institutional safety, collective efficacy, and supportive peer networks (Sampson et al., 1997). Depressive symptoms accumulate through chronic stress and eroded sense of control and may be less directly modifiable by school belonging alone. PTSS may be more directly mitigated by predictable and stable school environments, given that such settings can reduce perceived threat and foster a sense of safety (Ehlers & Clark, 2000).

Depressive symptoms, by contrast, may stem from broader structural stressors such as financial strain and family hardship that accumulate outside the school environment and are less directly modifiable by school belonging alone (Gilbert et al., 2017; Steare et al., 2023). It is worth noting that the absence of a buffering effect for depressive symptoms does not diminish the broader importance of school belonging as a developmental resource. Prior research has linked school belonging to a wider range of psychosocial outcomes beyond depression and PTSS, including developmental competence, externalizing behaviors, hope, future orientation, and overall well‐being (Eccles & Roeser, 2011; Gaias et al., 2019), suggesting that its protective function may operate most effectively for outcomes tied to behavioral regulation and future orientation rather than severe internalizing symptomatology, and across multiple domains that the present study was not designed to capture. These findings support a developmental perspective in which the psychological consequences of CVE are shaped not only by exposure severity but also by the availability of supportive contexts that can mitigate stress.

Patterns observed across the early adolescent and young adult samples suggest potential developmental differences in how youth respond to CVE and how school relationships are linked to these responses. Importantly, these differences should not be interpreted as contradictory findings, but rather as reflecting developmental variation in how school belonging functions within distinct ecological contexts. Consistent with the Social Ecological Model of Resilience (Ungar, 2011), these findings illustrate that the protective or reactive function of school belonging depends heavily on developmental stage and ecological fit. Early adolescents rely on school as a primary social and emotional environment (Eccles & Roeser, 2011), and when school‐based safety and support clash with violent community conditions, this ecological mismatch may heighten distress rather than buffer it. In contrast, young adults operate within broader ecological systems, including more flexible academic environments, expanding peer networks, and greater personal autonomy (Arnett, 2000), which may allow them to access and use school belonging as a meaningful resilience resource. These patterns highlight the importance of developmental timing within an ecological framework because the same exposure to violence can produce different psychological consequences depending on the alignment between youth's developmental needs and the supports available in their social ecologies. Taken together, these results underscore that interventions aiming to enhance school belonging must consider age, context, and ecological fit to avoid unintended reactive effects while promoting genuine resilience.

## LIMITATIONS

Several limitations should be considered when interpreting these findings. First, cross‐sectional design prevents inferences about temporal ordering or developmental change. Longitudinal work is needed to clarify whether school belonging becomes protective over time or whether the observed developmental differences reflect cohort‐specific contextual realities rather than age‐related processes. In a related vein, differences in demographic composition, sample size, and variability between the early adolescent and young adult samples may have resulted in differential statistical power, particularly for detecting higher order interaction effects. As such, differences in statistical significance across samples should not be interpreted as definitive evidence of developmental differences. These patterns highlight the importance of developmental timing within an ecological framework because the same exposure to violence can produce different psychological consequences depending on the alignment between youths' developmental needs and the supports available in their social ecologies.

Additionally, structural differences between the school environments included in this study (i.e., public middle schools vs. public university setting) may partly explain developmental differences in the role of belonging. School belonging represents one component of the broader school climate and may vary across institution‐specific characteristics such as teacher availability, class sizes, academic demands, and extracurricular opportunities. Future research should more fully capture the multidimensional nature of school climate, including safety, engagement, and institutional support, to better understand how these factors jointly shape youth outcomes. Lastly, the young adult sample was drawn exclusively from a public university, which may underestimate the burden of violence exposure among young adults who are not enrolled in higher education. University‐attending youth in this region likely represent a more socioeconomically advantaged and potentially more protected subgroup, which may have attenuated the observed effects.

## CONCLUSION

Taken together, findings from the current study underscore the developmental and contextual complexity of school belonging as a protective factor in high‐risk settings. Although school belonging was linked to lower psychological distress among early adolescents, this pattern did not extend into young adulthood, highlighting the need to consider how developmental stage, educational context, and sociocultural conditions jointly shape youths' experiences of safety and belonging. Future work that follows youth longitudinally, examines variation in school structures and resources, and incorporates additional relational and contextual supports will be essential for clarifying when, how, and for whom school belonging exerts its protective effects. Such efforts can advance developmental theory and inform policies aimed at strengthening educational environments for youth growing up amid chronic violence.

## AUTHOR CONTRIBUTIONS

**Andrea S. Medrano:** Conceptualization; investigation; funding acquisition; writing – original draft; methodology; writing – review and editing; formal analysis; project administration; supervision; resources; data curation. **Francheska Alers‐Rojas:** Project administration; supervision; data curation; writing – review and editing. **Patrick T. Quintero:** Conceptualization; writing – original draft; writing – review and editing. **Erika Álvarez Álvarez:** Supervision; project administration; writing – review and editing.

## FUNDING INFORMATION

This work was supported by the University of Pittsburgh Momentum Priming Grant and the Hewlett International Grant awarded to the first author.

## CONFLICT OF INTEREST STATEMENT

The authors declare that there is no conflict of interest regarding the research, authorship, and/or publication of this article.

## ETHICS STATEMENT

All procedures and materials for Study 1 and 2 were reviewed and approved by the Institutional Review Board (IRB) at the University of Pittsburgh. The initial study approvals were granted on June 12, 2024, under ID: STUDY23110043 (Study 1) and on May 20, 2024 under ID: STUDY23120079 (Study 2). Additionally, local authorization for all procedures, materials, and data collection was obtained and approved by Comisión de Ética on May 10, 2024 (Study 1 and Study 2) from Universidad Michoacana de San Nicolás de Hidalgo.

## CONSENT

Written parental consent and written youth assent were obtained for all participants in Study 1. For the young adult participants in Study 2, written informed consent was obtained prior to data collection. These consent procedures were implemented to ensure that all participants, and the guardians of those under the legal age of consent, were fully informed of the study's purpose and the voluntary nature of their participation.

## Data Availability

Data are available from the corresponding author upon reasonable request, in accordance with ethical and institutional guidelines.
